# Chinese Herbal Medicines and Conventional Chronic Heart Failure Treatment for the Management of Chronic Heart Failure Complicated with Depression: A Systematic Review and Meta-Analysis

**DOI:** 10.1155/2020/8627928

**Published:** 2020-04-21

**Authors:** Peidan Yang, Jun He

**Affiliations:** ^1^Guangzhou University of Chinese Medicine, Guangzhou 510405, China; ^2^Department of Rehabilitation Center, The First Affiliated Hospital of Guangzhou University of Chinese Medicine, Guangzhou 510405, China

## Abstract

**Background:**

Combination therapy with Chinese herbal medicines (CHMs) and conventional medical treatment (CMT) was proposed as a therapeutic strategy for chronic heart failure (CHF) patients complicated with depression. Therefore, we performed a systematic review and meta-analysis of randomized controlled trials (RCTs) to assess effects of this combination therapy on CHF complicated with depression.

**Methods:**

RCTs comparing the combination of CHMs and CMT to CMT were retrieved in seven electrical databases till March 30, 2019. The effective rate of cardiac function and Hamilton depression scale (HAMD) were marked as the primary outcomes. Left ventricular ejection fraction (LVEF) and Minnesota Living with Heart Failure Questionnaire (MLHFQ) were marked as the secondary outcomes. The methodological quality of eligible RCTs used the Cochrane bias risk assessment tool. Stata 13.0 and Review Manager 5.3 were preferred for synthesizing the results if the results were appropriate.

**Results:**

Thirteen RCTs enrolling 1022 subjects met the inclusion criteria. The majority of the retrieved RCTs were evaluated to be of low methodological quality. The pooled results of the meta-analysis showed that CHMs plus CMT group created better outcomes compared to CMT alone therapy, as evidenced by the fact that the overall effects of combination therapy strategy were significantly greater than the control group in increasing effective rate of cardiac function (risk ratio (RR) = 1.28; 95% CI: 1.16 to 1.42), in improving depressive symptoms (HAMD) (standard mean difference (SMD) = −1.31; 95% CI: −1.68 to −0.95) and quality of life (MLHFQ) (weighted mean difference (WMD) = −8.42; 95% CI: −10.08 to −6.76), in increasing LVEF scores (WMD = 5.33; 95% CI: 4.30 to 6.35).

**Conclusion:**

The combination of CHMs and CMT increased the effective rate of cardiac function and LVEF scores and reduced HAMD and MLHFQ scale scores, which was a potential therapeutic strategy that improved the management of CHF patients complicated with depression. Future trials were needed to verify the above results since abnormal heterogeneity and poor quality of literature have existed in the included studies.

## 1. Introduction

Chronic heart failure (CHF) was a group of chronic progressive syndromes developed from a variety of organic cardiac diseases that endangered the patient's physical and mental health, and depression was one of the most common psychological complaints [1]. Since the discrepancies in study design and definition of depression, a series of studies suggested that the incidence of depression in CHF was between 23% and 60% [2–4]. The latest meta-analysis showed that the incidence of depression in Chinese CHF patients was about 40.1% [5]. The existence of depression not only adversely affected clinical outcomes and prognosis in CHF patients [6] but also increased the rate of rehospitalization and mortality which contributed to the significant healthcare cost of this chronic disease and reduced the quality of life of patients [7]. CHF patients often experienced fatigue, insomnia, and other autonomic nervous functions. The overlap of these clinical features with depression led to a challenge in diagnostics, which delayed initiating appropriate antidepressant therapy. The low diagnosis and curative rate of depression might be one of the reasons for the high morbidity and mortality of CHF patients [8]. At present, the mechanism of depression affecting HF remained controversial. A series of studies indicated that behavioral risk factors such as smoking, obesity, lack of exercise, excessive use of antidepressants [7], pathophysiological factors such as fibrinogen thrombosis [9], abnormal hypothalamic-pituitary-adrenal (HPA) axis regulation [10, 11], and elevated inflammatory biomarkers [12, 13] contributed to bad influence on CHF with depression.

Since the conception of psychocardiology [14] has been put forward and the incidence of CHF complicated with depression gradually increased, it was no wonder that treatments for CHF complicated with depression multiplied over the years. Accumulating evidence has shown that antidepressants [15], psychotherapy [16, 17], exercise training [18, 19], and electroconvulsive therapy [20] effectively alleviated the symptoms of CHF and depression. Selective serotonin reuptake inhibitors (SSRI) [15] were currently the most recommended antidepressant, but the side effect and the expensive price severely restricted its applications in the cardiovascular field. Moreover, clinical trials showed that the use of antidepressants might not improve the symptoms and prognosis of CHF with depression as expected [18]. Recognizing that Chinese herbal medicines (CHMs) combined with conventional medical treatment (CMT) has been extensively used in clinical practice [21–23]. However, individual studies did not provide sufficient evidence and the role of CHMs for CHF patients with depression remained controversial. Therefore, we aimed to objectively assess the potential benefits of this combination treatment in the management of CHF patients complicated with depression through a systematic review and meta-analysis.

## 2. Methods

### 2.1. Registration

Following the preferred reporting items for systematic reviews and meta-analyses (PRISMA) guidelines [24], our manuscript has been registered with PROSPERO (no. CRD 42019134281) which was available online at https://www.crd.york.ac.uk/PROSPERO/display_record.asp?CRD42019134281.

### 2.2. Literature Strategy

A systematic literature search was conducted (from inception to March 30, 2019) using four international electronic databases (PubMed, EMBASE, Cochrane Library, and Web of Science) and three Chinese literature databases (CNKI, WANGFANG, and VIP). We identified relevant literature using the following search terms: [chronic heart failure OR CHF] AND [Chinese herbal medicines OR CHMs OR traditional Chinese medicine OR TCM] AND [depression OR depressive symptom]. We manually searched references to detect additional articles without language or publication restriction.

### 2.3. Eligibility Criteria

#### 2.3.1. Types of Studies

We regarded articles as eligible for inclusion if they were published randomized clinical trials (RCTs) done in CHF adults with depression.

#### 2.3.2. Types of Patients

Participants were 18 years and older and are clinically diagnosed with CHF complicated with depression, according to Guidelines for the Diagnosis and Treatment of Heart Failure in China, 2014 [25], New York Heart Association (NYHA) heart function classification standard revised in 1994 [26], and Diagnostic Criteria for Depressive Disorders in the American Psychiatric Association's Diagnostic and Statistical Manual of Mental Disorders, 5th Edition (DSM-5) [27].

#### 2.3.3. Types of Interventions

The combination treatment consisting of CHMs and CMT was tested as the intervention group, while the control group only received CMT, including angiotensin receptor blockers (ARBs), angiotensin-converting enzyme inhibitors (ACEI), *β*-receptor blocker, aldosterone antagonist, diuretic, digoxin, and ivabradine, which was recommended in 2014 Guidelines for Heart Failure in China [25]. CHMs included traditional formula, Chinese patent medicines, and Chinese medicine extract, irrespective of the mode of administration, dose, dosage form of prescription, and treatment duration.

#### 2.3.4. Types of Outcome Measures

Major outcomes are effective rate of cardiac function and Hamilton depression scale (HAMD). Secondary outcomes were left ventricular ejection fraction (LVEF) and Minnesota quality of life scale (MLHFQ).

### 2.4. Data Extraction

Two experienced investigators (Yang and He) independently browsed through the titles and abstracts of literature for removing irrelevant articles (pharmacokinetic studies, animal or laboratory studies, and general reviews) and duplication. Subsequently, studies that met inclusion criteria were retrieved for full-text evaluation. Abstracted data were extracted with a standardized form, including study characteristics (study design, author name, and publication year), patient characteristics (age, gender, and NYHA class), and a specific description of the experiment and control group (intervention, outcomes, duration, and adverse reaction). Divided opinions would be resolved by consensus and further discussion with a third reviewer.

### 2.5. Quality Assessment of Studies

The bias risk assessment tool recommended by the Cochrane Handbook for Systematic Review of Interventions [28] was applied to evaluate the methodological quality of RCTs through the following six domains: random sequence of generation, allocation concealment, blinding of participants and personnel, blinding of outcome assessment, incomplete data addressed, selective reporting, and other bias [28, 29]. The evaluation results were divided into three categories: high risk of bias, low risk of bias, and unclear risk of bias.

### 2.6. Statistical Analysis

Relative risk ratio (RR) with 95% CI was selected as the dichotomous outcomes, and weighted mean difference (WMD) with 95% CI was selected as the continuous data. Because depression measurements were performed using different versions of the HAMD, the results were combined into standard mean difference (SMD) with 95% CI. Heterogeneity was quantitatively analyzed in this study using *I*^2^, which was over 50% considering the significant heterogeneity. In sensitivity analysis, metaregression was performed to detect potential heterogeneity. To ensure the results were robust and reasonable, we excluded each trial by sensitivity analysis and compared it with previously unresolved results. If the statistical heterogeneity cannot be eliminated and *I*^2^ > 50%, the random effect model was employed. Otherwise, a fixed-effect model was applied. We constructed the funnel plot to evaluate the publication bias by using Egger's test, and if there was a publication bias (*P* < 0.1), the result was revised by the trim-and-fill computation. We used the Stata (version 15.0) and Review Manager (version 5.3) for statistical analyses and assessment of methodological quality. *P* < 0.05 was deemed statistically significant.

## 3. Results Search Flow

**Figure 1:**
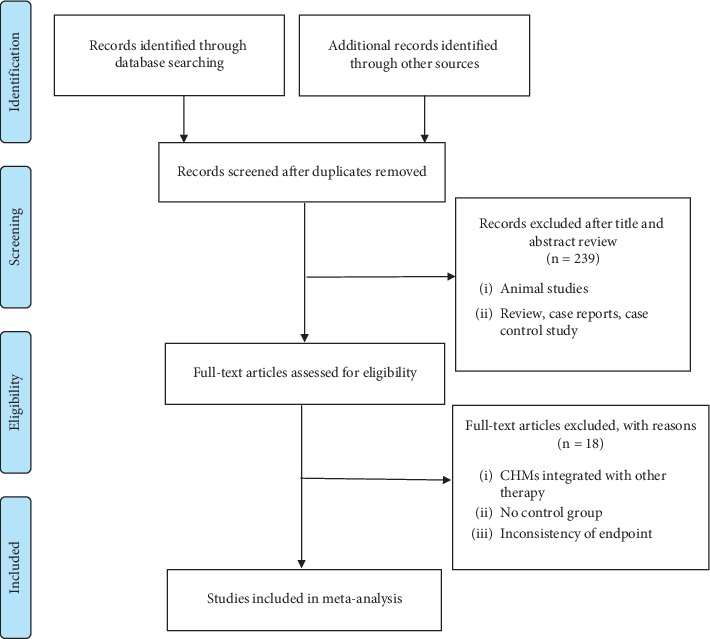
The ﬂowchart of study selection.

Figure 1 summarized the process of studies included in our analysis. We searched for 455 related studies and removed 185 repetitive articles. Subsequently, we excluded 239 trials that did not satisfy the conditions (studies were review or case reports or case-control study or animal studies) by screening the titles and abstracts. A total of 31 full-text studies were retested. After reading the full paper, 6 studies were with no control group, 4 articles were removed due to inconsistency of endpoint and quasi-randomized control trial, and 8 studies were CHMs integrated with antidepressants or physical therapy or psychological intervention. Therefore, thirteen [21–23, 30–39] studies met the inclusion criteria, and all of them were conducted in China.

### 3.1. Characteristics of Included Studies

Thirteen RCTs involved 1022 participants. Of these, 513 patients (50.2%) were treated with CHMs and CMT, and 509 patients (49.8%) were randomized to the CMT group. The sample size of patients in each study ranged from 40 to 155. Both men and women were included in our analysis, and more men were involved than women (55.7% males and 44.3% females). Over 80% of the patients were in NYHA classes II and III. Disease duration was reported in all trials ranging from four weeks to twenty-four weeks. Six studies reported no adverse reactions in the treatment of CHMs combined with CMT, while adverse reactions were not mentioned in five trials. Hua et al. [32] reported two cases of mild elevation of transaminase in the treatment group, which was reduced to normal after 8 days of reduced glutathione treatment. Wang et al. [35] reported four adverse reactions in the experimental group, including dry mouth in 1 case, constipation in 1 case, drowsiness in 1 case, and nausea in 1 case (Table 1).

### 3.2. The Methodological Quality of the Included Trials

**Figure 2:**
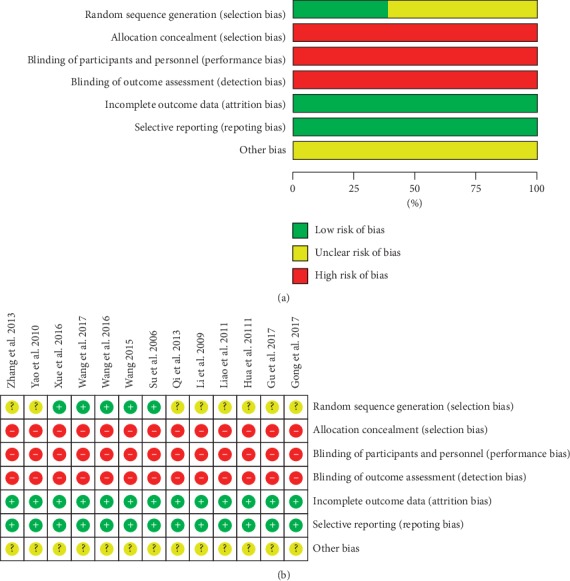
Methodological quality. (a) Risk of bias summary and (b) risk of bias graph.

All studies clearly stated that there was no significant difference between the two groups of baselines. In terms of selection bias, five [21, 31, 34–36] studies adopt a randomized digital table to generate random sequence, while eight [22, 23, 30, 32, 33, 37–39] trials merely mentioned “randomly” in their trials. One study [23] mentioned the condition of patients who had dropped out including six subjects in the experimental group and five subjects in the control group. In terms of performance and detection bias, none of the included studies manifested blinding of participants and personnel as well as an outcome assessment. Because of the lack of adequate allocation concealment, sample size calculation, a period of time to follow up after the intervention, and intentional analysis to process missing data, there were selection and other bias existing in all included trials. The majority of the evidence tended to be assessed as generally poor quality (Figure 2).

### 3.3. Primary Outcome

#### 3.3.1. The Effective Rate of Cardiac Function

**Figure 3:**
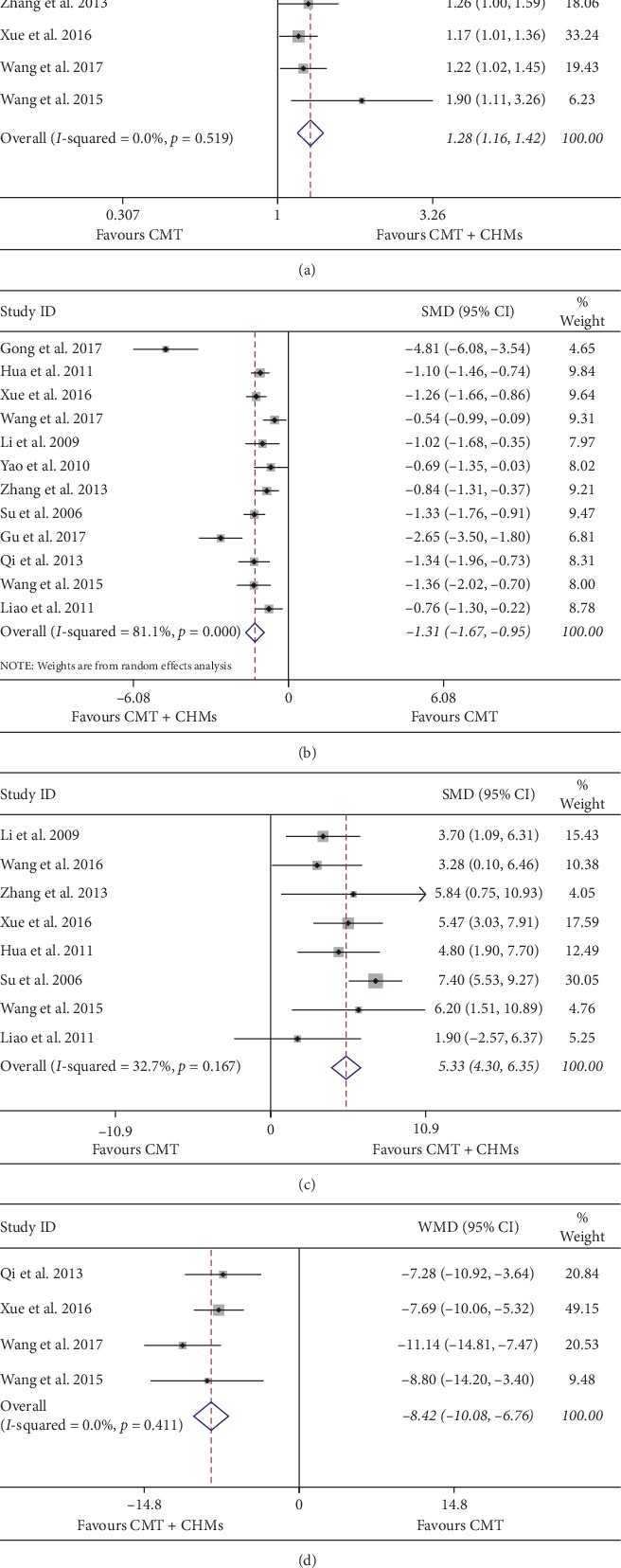
Forest plot of randomized controlled trials of CHMs and CMT therapy. (a) Effect of CHMs and CMT on cardiac function. (b) Effect of CHMs and CMT on symptoms of depression. (c) Effect of CHMs and CMT on LVEF. (d) Effect of CHMs and CMT on quality of life.

Pooled analysis of the six [21–23, 35, 36, 38] studies that assessed the improvement of curative effect on heart function when participants were treated with CHMs and CMT compared with CMT group, with difference between the two groups being statistically significant (RR = 1.28; 95% CI: 1.16 to 1.42, *P* < 0.001). No heterogeneity was observed between the studies (*P*=0.52, *I*^2^ = 0%) and a fixed-effect model was constructed (Figure 3(a)).

#### 3.3.2. Hamilton Depression Scale

**Figure 4:**
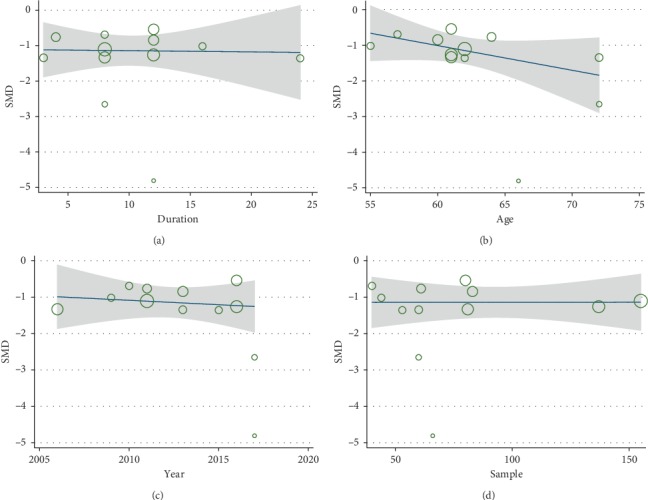
Metaregression of HAMD scale. (a) Treatment duration. (b) Mean age. (c) Publication year. (d) Sample size.

Due to the diversity of HAMD versions (HAMD-17, HAMD-21, and HAMD-24), we aggregated data on depressive symptoms by SMD with 95% CI. In a pooled analysis of twelve studies [21–23, 30–33, 35–39] using the HAMD scale to assess the depression, the combination of CHMs and CMT resulted in a greater reduction in the average degree of depression than CMT group, with difference between the two groups being statistically significant (SMD = −1.31; 95% CI: −1.68 to −0.95, *P* < 0.001). High heterogeneity was observed between the studies (*P*=0.001, *I*^2^ = 81.1%) and a random-effects model was constructed (Figure 3(b)). Therefore, we used sensitivity analysis and metaregression to seek possible sources of heterogeneity. Sensitivity analysis indicated that one trial [22] approximately increased overall heterogeneity by 20%. According to the characteristics of the study, we conducted a metaregression analysis one by one for the publication years, the sample size, treatment duration, and average age. The results showed that they were not the cause of heterogeneity (Figure 4).

### 3.4. Secondary Outcomes

#### 3.4.1. Left Ventricular Ejection Fraction

In a pooled analysis of eight [31–34, 36, 38] trials, the combination of CHMs and CMT led to a greater improvement in LVEF scores than CMT group, with the difference between the two groups being statistically significant (WMD = 5.33; 95% CI: 4.30 to 6.35, *P* < 0.001). A low heterogeneity was observed between the studies (*P*=0.17, *I*^2^ = 32.7%) and a fixed-effect model was constructed (Figure 3(c)).

#### 3.4.2. Minnesota Living with Heart Failure Questionnaire

Pooled analysis of the four studies [21, 30, 35, 36] used MLHFQ score to assess the improvement of quality of life (Qol) when participants were treated with CHMs and CMT compared with CMT therapy alone, with difference between the two groups being statistically significant (WMD = −8.42; 95% CI: −10.08 to −6.76, *P* < 0.001). There was no heterogeneity observed between the studies (*P*=0.41, *I*^2^ = 0%) and a fixed-effects model was constructed (Figure 3(d)).

#### 3.4.3. Sensitivity Analysis

**Figure 5:**
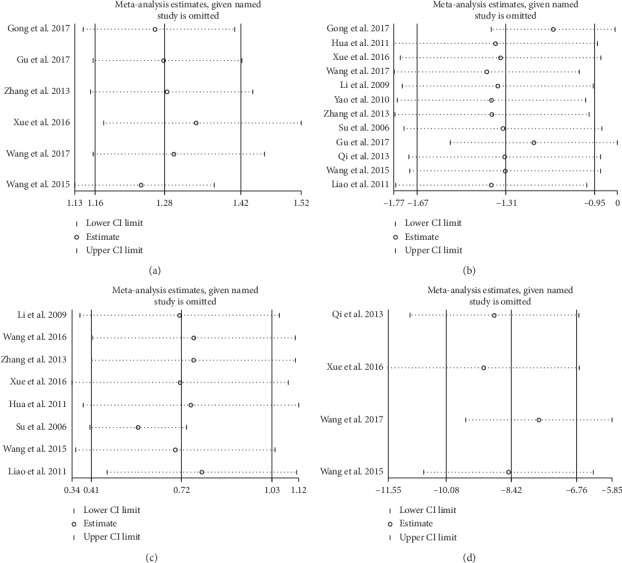
Sensitivity analysis of randomized controlled trials of CHMs and CMT therapy. (a) Effect of CHMs and CMT on cardiac function. (b) Effect of CHMs and CMT on symptoms of depression. (c) Effect of CHMs and CMT on LVEF. (d) Effect of CHMs and CMT on quality of life.

Sensitivity analysis was performed on the results of the effective rate of cardiac function, HAMD scale, LVEF score, and MLHFQ score. By eliminating each trial of each result one by one, the heterogeneity of each trial didn't exceed the 95% confidence interval, indicating that the overall results of our study were reliable and robust (Figure 5).

#### 3.4.4. Publication Bias

**Figure 6:**
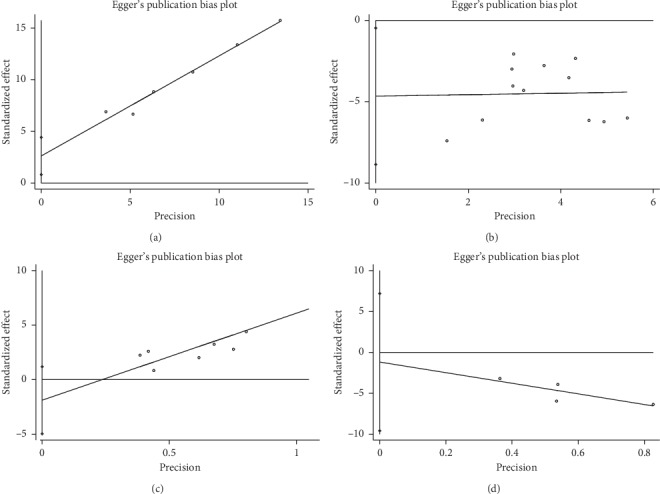
Egger's publication bias plot. (a) Effect of CHMs and CMT on cardiac function. (b) Effect of CHMs and CMT on symptoms of depression. (c) Effect of CHMs and CMT on LVEF. (d) Effect of CHMs and CMT on quality of life.

**Figure 7:**
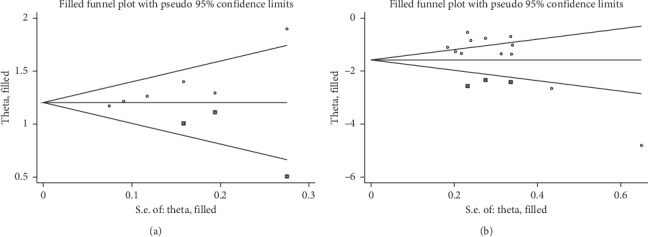
Filled funnel plot. (a) The effective rate of cardiac function. (b) HAMD scale.

Egger's test showed that there was no publication bias in terms of LVEF score (*P* > |*t*|=0.20, 95% CI = −4.97 to 1.20) and MLHFQ score (*P* > |*t*|=0.19, 95% CI = −20.71 to 7.73). Obvious publication bias was observed in terms of the effective rate of cardiac function (*P* > |*t*|=0.02, 95% CI = 0.83 to 4.42) and HAMD score (*P*=0.03, 95% CI = −8.85 to 0.46) (Figure 6). Subsequently, we used the trim and filling method to assess the reliability and stability of the effective rate of heart function and HAMD scale affected by significant publication bias. For the effective rate of cardiac function, the result showed that the RR and 95% CI after trim and filling method (RR = 1.25; 95% CI: 1.15 to 1.34; *P* < 0.001) were similar with the original result (RR = 1.20; 95% CI = 1.12 to 1.29; *P* < 0.001), indicating that the result was stable without flip. For the HAMD scale, the result demonstrated that the SMD and 95% CI after trim and filling method (SMD = −1.31; 95% CI = −1.68 to −0.95; *P* < 0.001) was consistent with the previous result (SMD = −1.58; 95% CI = −2.20 to −1.18; *P* < 0.001), indicating that the result was stable and reliable (Figure 7).

## 4. Discussion

### 4.1. Summary of Results

The meta-analysis showed that combination treatment consisting of CHMs and CMT effectively improved depressive symptoms and quality of life, increase effective rate of cardiac function and LVEF scores compared with CMT group alone. The promising evidence indicated that CHMs plus CMT might be beneficial to CHF patients with depression, at least in part, which supported the significant effect of integrated Chinese and Western medicine therapy. Nevertheless, the evident heterogeneity was observed in our analysis and we used metaregression, sensitivity analysis to explore the source of heterogeneity. Sensitivity analysis observed that one trial (Gong 2017) was one of the sources of heterogeneity and it might be caused by different HAMD versions and inconsistent outcome measures. Metaregression discovered that the publication year, sample size, course of the disease, and mean age were not the sources of heterogeneity. Relatively small sample size and possible clinical heterogeneity such as diversity of traditional Chinese medicine (various preparations, dosage, and syndrome classification) existing in our analysis and it would result in significant heterogeneity were of particular note.

Egger's test showed there was no publication bias in terms of LVEF score and MLHFQ score and then we performed a trim and filling method for the effective rate of cardiac function and HAMD scale. The combined results and 95% CIs did not change significantly and the funnel plot of them became symmetrical when three similar studies were added after using the trim and filling method, indicating that the conclusion was steady. We should consider the possibility that publication bias may result from language bias and exaggerated estimates due to a poor methodological design in some included trials.

The theory of traditional Chinese medicine (TCM) believed that the diseased region of CHF complicated depression was mainly in the liver, involving the heart and spleen. With the development of the disease and the prolongation of the disease, pathological changes of blood stasis would occur. Rules were mainly dispersing the liver, relieving depression, activating blood, and promoting diuresis. In the thirteen included trials, eight [22, 23, 30, 32–34, 38, 39] of them explicitly mentioned that the syndrome was stagnated liver and blood stasis, and dredging liver to regulate qi and remove stasis was adopted. A previous study demonstrated that herbals for promoting blood circulation and removing stasis together with regulating qi and relieving depression can promote metabolism and improve the excitability of the cerebral cortex and the depression state [40].

### 4.2. Subgroup Analysis (Mean Age and Treatment Duration)

To determine the optimal treatment strategy of CHMs and CMT, we performed a subgroup analysis of depression outcomes based on treatment duration and mean age (Table 2). The beneficial effect of CHMs plus CMT on the depressive symptoms was observed in both relatively young patients (mean age < 65 years) experiencing CHF with depression (three trials, random effects SMD = −1.01, 95% CI: −0.81 to 1.20, and *P* < 0.001; *I*^2^ = 29.7%) and in the elder (mean age > 65 years, nine trials, random effects SMD = −2.86, 95% CI: −4.63 to −1.08, and *P* < 0.001; *I*^2^ = 91.9%).

When limited to less 8-week treatment intervention, CHMs combined with CMT therapy demonstrated obvious reductions in depressive symptoms (3 trials, random effects SMD = −0.93, 95% CI: −1.33 to −0.53, and *P* < 0.001; *I*^2^ = 24.1%). The positive effect lasted from 8 to 12 weeks after the intervention (seven trials, random effects SMD = −1.57, 95% CI: −2.14 to−1.00, and *P* < 0.001; *I*^2^ = 88.7%) and over 12 weeks after the program (two trials, random effects SMD−1.19, 95% CI: −1.66 to −0.72, and *P* < 0.001; *I*^2^ = 0).

### 4.3. The Advantages and Mechanism of CHMs

The advantages of CHMs were as follows: firstly, whether single Chinese medicine, compound, or Chinese patent medicine, which not only improved physical function and psychological state but also improved Qol and caused minimal adverse reactions, secondly, the antidepression effect of CHMs being multitarget and multimechanism especially for patients with many symptoms and a long course of the disease, and, thirdly, doctors often using drugs flexibly based on the specific condition of patients, with the characteristics of individualized medication. There was some evidence to support the view that CHMs were safe and had better effects on depression than the placebo group. Previous review and meta-analysis indicated that St. John's wort extract, the main component of Shuganjieyu capsule, was significantly better than placebo in patients with mild to moderate depression, and it had the same benefits but fewer side effects than conventional antidepressants [41–43]. A multicenter, randomized, double-blind, and placebo parallel controlled trial showed that Qili Qiangxin capsules in patients with CHF significantly reduced NT-proBNP levels compared to that placebo [44]. Moreover, the Qili Qiangxin capsules outperformed the placebo on LVEF, Qol, and 6-minute walking distance (6-MWT) [45].

The therapeutic mechanism of CHMs for treating CHF complicated with depression has been investigated in a series of clinical trials and animal experiments at different stages. Previous studies demonstrated that CHMs had antieffect on depressive disorder, which might work by regulating the monoamine neurotransmitters and their receptor (norepinephrine, dopamine, and 5-hydroxytryptamine) [46, 47], HPA axis (serum corticotropin-releasing hormone, cortisol, and adrenocorticotropic hormone) [48, 49], neurotrophic factors and protective neurons in the hippocampus (nerve growth factor, brain-derived neurotrophic factor) [50–52], proinflammatory cytokines (interleukin-1*β*, interleukin-6, tumor necrosis factor-*α*, and interleukin-10) [53, 54], and intestinal flora (lactic acid bacteria and bifidobacteria) [55–57]. Some research suggested that CHMs had a significant effect on the improvement of cardiac function and mechanism was perhaps realized by strengthening myocardial contractility, increasing urine production, improving myocardial energy metabolism, protecting endothelial function, promoting angiogenesis, inhibiting overactivation of neuroendocrine system, and slowing ventricular remodeling (inflammatory response, myocardial fibrosis, apoptosis, and autophagy) [58–61].

### 4.4. Strengths and Limitations

This paper was the first systematic review and meta-analysis to assess and compare the effects of the combination of CHMs and CMT with CTM alone regarding curative rate of heart function, HAMD scale, LVEF scores, and MLHFQ scores among CHF patients with depression. Although the results were encouraging, there were several limitations to our analysis. Almost all participants involved in this analysis were diagnosed with NYHA class II and class III, so our results might not be appropriate for severe CHF with depression. Depression included mild depression, moderate depression, and severe depression, but we did not perform subgroup analysis due to incomplete descriptions. According to differentiation treatments on the different syndrome characteristics, different types of prescriptions were used and thus there was obvious discrepancy between the individual trials, which may be led to clinical heterogeneity existing in our analysis. Besides, we considered that CHMs involving some behaviors, such as going to the doctor, obtaining a prescription, and taking it on a doctor's advice, potentially influenced the therapeutic outcome.

Regarding the particularity of CHMs, our analysis was mainly conducted in China and all results should not be extrapolated to other populations outside of Asia, and further trials in different population to strengthen the validity of the evidence were urgently needed. The evidence was based on data on short-term efficacy, while the long-term efficacy data was insufficient. We found that some trials did not report adverse events in individuals, and we were unable to assess the balance of harm and benefit in the general population using this combination therapy. CMHs included different prescriptions and dosages, and it was critical to perform detailed subgroup for various prescriptions and different dosages in the future. The existing CHMs studies were almost based on clinical trials with incomplete design, small sample, relatively short intervention, and inconsistent efficacy evaluation, and therefore it's urgent to conduct prospective, well-designed RCTs to increase our knowledge.

For the limitations and deficiencies of the existing studies, it is suggested that future research should emphasize the design of studies, especially the detailed description of random sequences, the attention of allocation concealment, and intention analysis as well as blinding. It is necessary to enlarge the sample size and prolong the intervention time and provide sufficient follow-up period. Establishing a domestic or even international syndrome classification standard is beneficial for clinical guidance. The methodological quality of the included RCT trials needs to be improved, which may have a potential influence on the outcome of the intervention. Considering the characteristics of CHMs, it's hard to blind patients but we can blind researchers who are responsible for evaluating and recording results.

## 5. Conclusions

This study indicated that CHMs plus CMT could be more beneficial than CMT alone for increasing curative rate and LVEF scores, improving depressive symptoms and Qol in CHF patients with depression. Therefore, our meta-analysis and systematic review results will be helpful for patients, medical personnel, and clinical decision-maker. Unfortunately, the methodological quality of literature was generally low and heterogeneity broadly existed in our analysis. Though there are several relevant trials about CHMs in the treatment of CHF with depression in progress, we look forward to the multicenter randomized controlled trials with large sample sizes, rigorous design, standardized outcomes, and long follow-up period that will be carried out in the future.

## Figures and Tables

**Table 1:** Baseline characteristics of included studies.

Author	*N*		Gender		Age (years)		NYHA	Intervention		Duration (weeks)	Adverse reaction	Outcome
	*T*	*C*	*M*	*F*	*T*	*C*	Class	*T*	*C*
Gong et al. 2017	33	33	41	25	66.5 ± 7.8	66.8 ± 7.2	II∼III	Sini powder + CMT	CMT	12	No	Effective rate of cardiac function/HAMD-24
Li et al. 2009	22	22	23	21	55.1 ± 6.8	54.6 ± 6.6	II∼III	Xiaoyao powder + CMT	CMT	16	No	HAMD-17/LVEF
Hua et al. 2011	78	77	89	66	62.7 ± 4.6		III∼V	Shugan jieyu capsule + CMT	CMT	8	Yes	HAMD-24/LVEF
Yao et al. 2010	20	20	29	11	58.9 ± 6.83	56.4 ± 6.71	II∼III	Xinshu granules + CMT	CMT	8	NR	HAMD-17
Qi et al. 2013	30	30	34	26	72.23 ± 6.56	73.07 ± 6.52	II∼IV	Yixin decoction + CMT	CMT	4	NR	HAMD-21/MLHFQ
Wang et al. 2017	30	30	34	26	68.50 ± 3.03	69.27 ± 3.29	II∼IV	Kaixin powder + CMT	CMT	4	No	Effective rate of cardiac function/MLHFQ
Zhang et al. 2013	43	40	55	28	59.31 ± 10.19	61.0 ± 8.39	II∼IV	Qili qiangxin capsules + CMT	CMT	12	No	Effective rate of cardiac function/LVEF/HAMD-17
Xue et al. 2016	69	68	75	62	61.9 ± 10.8	62.6 ± 11.3	II∼III	Zhenwu decoction with xiaoyao powder + CMT	CMT	12	No	Effective rate of cardiac function/HAMD-24/LVEF/MLHFQ
Wang et al. 2016	39	42	28	53	61.32 ± 15.34	62.31 ± 14.09	II∼IV	Shuxin soup + CMT	CMT	12	NR	Effective rate of cardiac function/HAMD-24/LVEF
Su et al. 2006	62	61	80	43	61.3 ± 15.34	62.31 ± 14.09	II∼III	Wuling powder + CMT	CMT	8	NR	HAMD-17/LVEF
Gu et al. 2017	30	30	27	33	72.6 ± 6.1	72.3 ± 5.6	I∼III	Yixin jieyu decoction + CMT	CMT	8	No	Effective rate of cardiac function/HAMD-17
Wang et al. 2015	26	26	31	21	63.18 ± 12.46	62.35 ± 11.65	II∼III	Shuganjieyu capsules + CMT	CMT	24	Yes	HAMD-17/MLHFQ/LVEF
Liao et al. 2011	31	30	25	36	64.2 ± 12.5		≥II	Shuganjieyu capsules + CMT	CMT	4	NR	HAMD-17/LVEF

CMT: Conventional medicine treatment; NR: no report; HAMD: Hamilton depression rating scale; LVEF: left ventricular ejection fraction; MLHFQ: The Minnesota Living with Heart Failure Questionnaire.

**Table 2:** Subgroup analysis for treatment duration and mean age.

Depression outcome total or subgroup	Trials	Effects model	Pooled effect	95% CI	*P* value	*I* ^2^ (%)
Total	12	Random	SMD−1.31	−1.68, −0.95	<0.001	81.1
Total (remove Gong et al.)	11	Random	SMD−1.12	−1.37, −0.87	<0.001	61.3
Mean age						
<65 years	3	Random	SMD−1.01	1.20, −0.81	<0.001	29.7
>65 years	9	Random	SMD−2.86	−4.63, −1.08	0.02	91.9
Treatment duration						
<8 weeks	3	Random	SMD−0.93	−1.33, −0.53	<0.001	24.1
8–12 weeks	7	Random	SMD−1.57	−2.14, −1.00	<0.001	88.7
>12 weeks	2	Random	SMD−1.19	−1.66, −0.72	<0.001	0
